# Bipolarity and suicidal ideation in children and adolescents: a systematic review with meta-analysis

**DOI:** 10.1186/s12991-017-0143-5

**Published:** 2017-04-21

**Authors:** Flórido Sampaio das Neves Peixoto, Danilo Ferreira de Sousa, Dayse Christina Rodrigues Pereira Luz, Nélio Barreto Vieira, Jucier Gonçalves Júnior, Gabriel Cabral Alencar dos Santos, Flaviane Cristine Troglio da Silva, Modesto Leite Rolim Neto

**Affiliations:** 1 Program in Health Sciences, ABC School of Medicine-FMABC, Santo André, SP Brazil; 2 Nursing Student at School of Juazeiro do Norte-FJN, Juazeiro do Norte, CE Brazil; 3 School of Medicine, Federal University of Cariri (UFCA), Divino Salvador Street, 284, Rosário, Barbalha, CE 63180-000 Brazil; 4 School of Medicine, Estácio-FMJ, Juazeiro do Norte, CE Brazil

**Keywords:** Adolescent, Bipolar disorder, Child, Mental health, Risk factors, Suicidal ideation

## Abstract

**Background:**

Affective disorders in children and adolescents have received growing attention in the world scenario of mental health. Additionally, there has been an increasing prevalence of suicidal ideation in this population.

**Objective:**

A systematic review with meta-analysis was conducted to demonstrate the main risk factors regarding the development of suicidal ideation in the bipolar disorder.

**Methods:**

This is a systematic review with meta-analysis using the PRISMA protocol (http://www.prisma-statement.org/). This study included secondary data. Original data in mental health were collected by mapping the evidence found in the following electronic databases: MEDLINE/PubMed, LILACS, SciELO, and ScienceDirect in the period from 2005 to 2015.

**Results:**

We found 1418 registrations in such databases, and 46 of them were selected to comprise this review. The result introduces a joint risk between the studies of 2.94 CI [2.29–3.78]. A significant correlation was verified between the risk factors and the suicidal ideation. The result was *r* (Pearson) = 0.7103 and *p* value <0.001.

**Conclusion:**

Children and adolescents living with bipolar disorder are more vulnerable to suicidal ideation. These results reinforce the need of a more effective public policy directed toward this population.

## Background

The bipolar disorder (BD) is a chronic and recurrent mental disturbance that can result in functional and cognitive impairment for the subject if not treated effectively. It has higher prevalence in adults; however, it can also affect children and adolescents. Nevertheless, the manifestation in such population is different in adults, who usually show the classical pattern of the pathology [1]. These subjects can manifest behavioral variations, such as mania (euphoric, fearless, and hyperactive behavior) or depression (weepy, sad, and isolated behaviors, among others) [2].

Gilman et al. [3] emphasize that children who have gone through adversities and stressful episodes, like financial difficulties in the family, maltreatment, and sexual abuse in this life phase, have 1.5–3 times higher chances of developing the BP. The authors verified this association statistically and demonstrated an odds ratio (OR) of around 2.23 for abuse; and 2.10 for maltreatment. This association was statistically significant for recurrent mania, with an OR of 1.55 for abuse; and 1.60 for maltreatment.

Braga and Del’Agio [4] found stressful events or exposure to violence, like excessive use of licit or illicit substances, economical or emotional (losses or separations) family problems, as the main risk factors for BD in adolescence. With regard to gender, girls usually try to commit suicidal ideation more often than boys; however, the latter have a higher rate of success, since they usually choose more violent ways to commit suicidal ideation.

Hence, human self-destructive behavior is a reality that affects many countries in the world. It is a public health issue of high complexity, multifactorial causes, and great socioeconomic and family impact [5]. Alves Jr. et al. [6] conducted a study with 1132 adolescents aged 14–19 years from public schools in the city of São José (SC, Brazil). A prevalence of 13.8% was found for suicidal thinking, a 10.5% for planning, and a 5.5% for suicidal ideation attempts. There was a higher rate of males (54.2%); 61.8% of the participants were white-skinned and 70.0% had a high socioeconomic level. Another important factor was the sleep pattern of these adolescents: about 75% presented a disturbed sleep pattern, i.e., they did not sleep well. This population showed the highest rates regarding suicidal thinking (22.7%), planning (17.2%), and attempt of suicidal ideation (9.4%); therefore, it was statistically significant (*p* < 0.01).

In turn, the map of violence disclosed in 2015 highlights the suicidal ideation rate of 16- to 17-year-old adolescents in Brazil, which increased to 80.8%. A retrospective study was conducted to catalog and analyze data from 1980 (156 cases) to 2013 (282 cases) [7]. Children and adolescent’s suicidal ideation is a reality and has had a growing incidence [8].

The BD is a pathology that can cause other psychic comorbidities like anxiety and eating disorders, affecting 42 and 17% of this population, respectively [9], as well as physical disorders like diabetes mellitus [10]. This fact can cause the feeling of conflicting emotions or self-perception alterations that may result in suicidal ideation. Costa [11] emphasizes that subjects who live with BD present a higher prevalence of psychic and/or physical comorbidities and therefore have an increased risk of suicidal ideation.

Based on these facts, we have noticed the further need of studying such theme, which was possible through a systematic review with meta-analysis to identify the statistical significance of the association factors between the BD and the suicidal ideation in children and adolescents.

A guiding question was created for the research in order to achieve such objective following the PICO acronym, in which each letter represents a component of the question, according to the concepts: P—adolescents and children; I—bipolar disorder; C—without disorder; and O—suicidal ideation. The guiding question was based on the questioning about the statistical significance seen through the association factors between the BD and the suicidal ideation—when it had been analyzed on the standpoint of children and adolescents.

This strategy allowed making the following question of research: are the factors of association between the BD and the suicidal ideation in children and adolescents statistically significant?

## Methods

This is a systematic review with meta-analysis that used the PRISMA protocol (http://www.prisma-statement.org/). The study objective, record eligibility criteria, interpretation methods, and outcome analysis were defined before the study.

This study included original analyses based on secondary data from the World Health Organization (WHO), the Department of Health and Human Services—UK, the National Epidemiological Catchment Area Study (ECA/USA), and the Brazilian Ministry of Health. The information provided in these systems is available online in electronic databases.

The search for original data about mental health was filtered by mapping the evidence found in the following electronic databases: MEDLINE^®^/PubMed, LILACS, SciELO, and ScienceDirect in the period from 2005 to 2015. The time frame was based on the significant development regarding knowledge of bipolar affective disorder (BAD) in childhood and adolescence for the last 15 years.

The analysis comprised the following keywords in Portuguese and English (DeCS and MeSH databases): #1. “bipolar disorder,” #2. “suicide,” #3. “child,” and #4. “adolescent.” The Boolean operators “AND” and “OR” were used: #1 and #2 and #3 or #4.

Access to gray literature was done through hand searching to actively identify eligible studies that were not retrieved in the search strategy. Gray literature was used to provoke theoretical interfaces that are reliable to what is upon the relevant information regarding government reports and/or documents.

We included epidemiological, population-based, observational, longitudinal, cross-sectional, and case–control studies, in Portuguese and English, from 2005 to 2015. We excluded studies referring to suicidal ideation in a superficial manner that did not mention its possible associated factors or that did not have a clear positioning on the mentioned subject, in the authors’ opinion. Two reviewers independently analyzed the scientific evidence according to the established criteria. In case of disagreement, a third reviewer was consulted to make a decision on the inclusion of studies or even on the reliable interpretation of data.

BioEstat 5.0 program was used for statistical analysis and to measure the risk of a child or adolescent developing suicidal ideation. Pearson’s correlation was calculated to verify the strength of the statistical relation between the evaluated factors and the outcome of patients in suicidal ideation development.

## Results

**Fig. 1:**
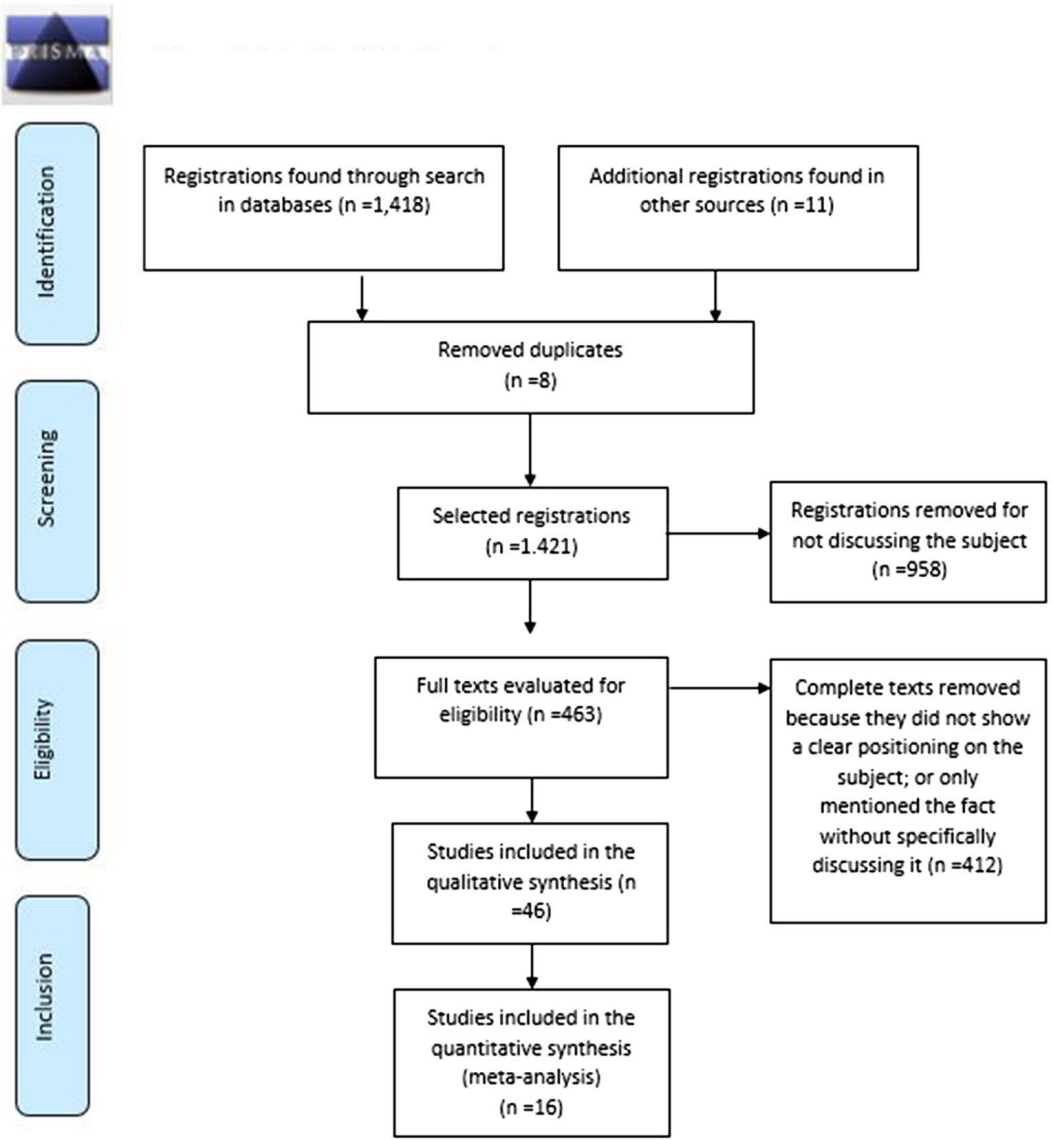
Flowchart of study search

We found 1418 registrations in the databases: 9 at SciELO; 1053 at ScienceDirect, and 356 at PubMed/Medline. The scanning of the title and abstract resulted in 463 articles. The detailed reading of studies through the full text and confirmation of eligibility resulted in 42 articles. After adding other 4 documents of gray literature, a total of 46 pieces of evidence was found. Of these registrations, 16 articles were inserted in the meta-analysis (Table 1). Figure 1 shows the methodological process of evidence search to be the basis of the systematic review and meta-analysis.

**Table 1:** Definitions of bipolar disorder according to the literature

Author	Definition
Mason [12]	Spectrum that emphasizes two conditions: the lowest, melancholia; and the highest, mania
Koenders et al. [29]	BD is a chronic and highly disabling disease that is characterized by the constant risk of recurrence
Kerner [30]	Bipolar disorder is a complex genetic disorder; however, it has not been discovered how it is transmitted yet. Many investigators believe that common genomic variants present a risk of disease manifestation
APA [31]	It consists in one or more maniac or mixed episodes, with frequent presentation of one or more depressive episodes. If it is BAD I, there is at least one hypomania episode associated and, at least, one longer depressive episode, if it is BAD II
Meier et al. [32]	Severe psychiatric condition characterized by fundamental and distinctive distortions of emotion and perception regulation
Alvarez [33]	The BAD is characterized by humor alterations, alternating between maniac and depressive episodes, which can have different intensity and duration according to personal and situational characteristics
Lee [34]	Although there are still some disagreements regarding the clinical characteristics and doubts on which would be the cardinal symptoms of the disorder in children and adolescents, the world testified an amazing increase of BD incidence in this population during the last decade. Some studies suggest that the phenomenology of humor disorders can vary based on the cognitive functioning, social ability, and degree of psychological development of each subject
Department of Health and Human Services [35]	Disorder that causes non-usual alterations of humor and that can cause damage at school or even in interpersonal relationships
Abreu et al. [36]	Disorder that is strongly associated with suicidal ideation, suicide attempts, and suicide itself
Miasso [37]	Chronic disorder characterized by the existence of acute and recurrent episodes of humor pathological alteration
Costa [11]	Recurrent, chronic, and severe disease with significant impact on patients’ quality of life, and it is also a great burden to the family and society in general
OMS [38]	Disorder characterized by two or more episodes, in which the subject’s humor and level of activity are deeply disturbed. And such disturbance consists in some occasions of a humor rise and increase of energy and activity (hypomania or mania) and, in other times, a decrease of humor and energy and activity (depression)
Rocca and Lafer [39]	One of the most severe kinds of mental disease characterized by the presence of alternating episodes of humor (mania/hypomania and depression), which vary in intensity, duration, and frequency. Besides the classical episodes of mania, hypomania, and depression, mixed episodes, i.e., episodes with mania/hypomania phases and depression are also present
Vieira et al. [40]	The BHD is a severe, incurable, and cosmopolite disease. It is considered a complex disease that presents different clinical pictures and many neurobiological and etiological models that aim at explaining the appearance and manifestation of the disease
Moreno et al. [41]	Long-term, episodic, and potentially severe humor disorder that many times can have psychotic symptoms too. A continuous medical condition, for the entire lifecycle, with recurrent episodes that have great impact on the patient’s life, thus decreasing its functioning and quality of life

*BD* bipolar disorder, *BAD* bipolar affective disorder, *BHD* bipolar humor disorder

### Consequences of bipolar affective disorder

The BAD is a pathology characterized by alternating episodes of depression and mania or hypomania. In the depressive phase, the subject shows depressed humor, low self-esteem, and considerable attention deficit, whereas his/her humor is exalted, whether of joy or irritation, in the maniac episode. A feeling of indestructability, increase of physical vigor, and disinhibition are also common during this phase [1].

Although the BAD frequently affects adults as a possible indicator of the appearance of the first symptoms in the age of 20 years, research has showed its insertion in children and adolescent’s environments, since the age of 4 [11].

Comorbidity is one of the main reasons for the increase of burden and costs associated with the BAD. The main damage related to adolescents affected by this disease includes the deficit in social cognition, the excessive use of substances, and the suicidal ideation. These factors have a negative burden in treatment adhesion, higher frequency of exposure to risk situations, and death. Social cognition is the neurobiological process that allows a proper interpretation of social signs and proper conduction of our behavior before the society [12].

Brain structures involved in the control of social conducts include the ventromedial prefrontal cortex, in charge of social rationale and decision-taking; the tonsil, responsible for recognition of emotions in faces; right somatosensorily cortex, which regulates the emphatic behavior and simulation; islet, in charge of the autonomic response [13]. There is evidence of alterations in the volume of some of these structures in some mental pathologies [14]. Harmony between these brain regions enables proper conducts of adaptive value, thus providing the subject with a pathology-free condition and consequently allowing a satisfactory social living.

When the child grows unexpectedly, he/she learns to interpret and manipulate his/her emotions based on rules and expectations of the society, developing a correct social cognition [13]. However, a compromise of the evolution of cognitive spaces is seen in children and adolescents with BAD, because humor alternations negatively interfere in their psychosocial adjustment, thus signalizing compromise in the interface between “me” and the “world.” Research highlights decision-taking deficits; therefore, it causes a more frequent exposure of children and adolescents to risky situations like use of drugs, bad behavior, and opposition to authorities. It is worth noting that the reduction in the emotion recognition capacity causes important problems in the child’s modulation of provoking and aggressive behavior, thus creating violence or exclusion situations [14].

Hence, the decision-taking loss regarding the child causes oscillations between impulsivity and immediate view of things, as well as accommodation and repetition of behaviors in the depressive phase [15]. According to the ECA, the prevalence throughout life of substance abuse or dependence among patients with BAD is of 56% [16]. With regard to adolescents, a 16% prevalence was found among bipolar subjects [17]. In a study conducted with adolescents that sought treatment due to the use of substances in *Hospital das Clínicas* at the School of Medicine from *Universidade de São Paulo*, humor disorders were present before the beginning of drug use in 44% of the girls and in 12% of the boys [18]. Based on the observation of previously presented data, it is possible to infer that the presence of BAD in children and adolescents is a risk factor for the use of psychoactive substances. They contribute to worsening of the mental disorder condition due to humor alteration, whether it is during intoxication or during the abstinence phase or even due to the decrease of treatment adhesion [19]. Table 2 includes the main risk factors for the development of suicidal ideation in the BD.

**Table 2:** Risk factors for bipolar disorder

Author (year)	Risk factors
Holtzman et al. (2015) [18]	Family history and use of substances like alcohol
Breen et al. (2015) [25]	Child abuse and hypothalamic–pituitary–adrenal gene
Lan et al. (2015) [21]	Symptoms like low control and fast thoughts
Weinstein et al. (2015) [19]	Depression symptoms, quality of life, despair, self-esteem, and family strictness
Monfrim et al. (2014) [42]	Immunological dysfunction
Moor et al. (2012) [24]	More prevalent above 15 year old and associated with panic disorder
Goldstein (2009) [22]	Female gender, previous history of bipolar disorder, other psychological disorders, family genetics
Goldstein et al. (2005) [43]	Family history of suicide attempt, hospitalization history, and physical and/or sexual abuse history
Bernegger et al. (2015) [44]	Females, emotional and physical negligence and sexual and emotional abuse
Rajewska-Rager et al. (2015) [45]	Level of disease compromise
Ellis et al. (2014) [27]	Adjustability and family cohesion
Park et al. (2013) [21]	Depressive symptoms like discouragement and pessimism
Goldstein et al. (2012) [28]	Females, depressive symptoms, and family history of suicide
Singh and Coffey (2012) [46]	It can occur since the age of 5
Goldstein (2009) [22]	Family stress
Jolin et al. (2007) [23]	Clinical course, psychiatric comorbidities, familiar suicidal behavior, psychosocial factors

**Fig. 2:**
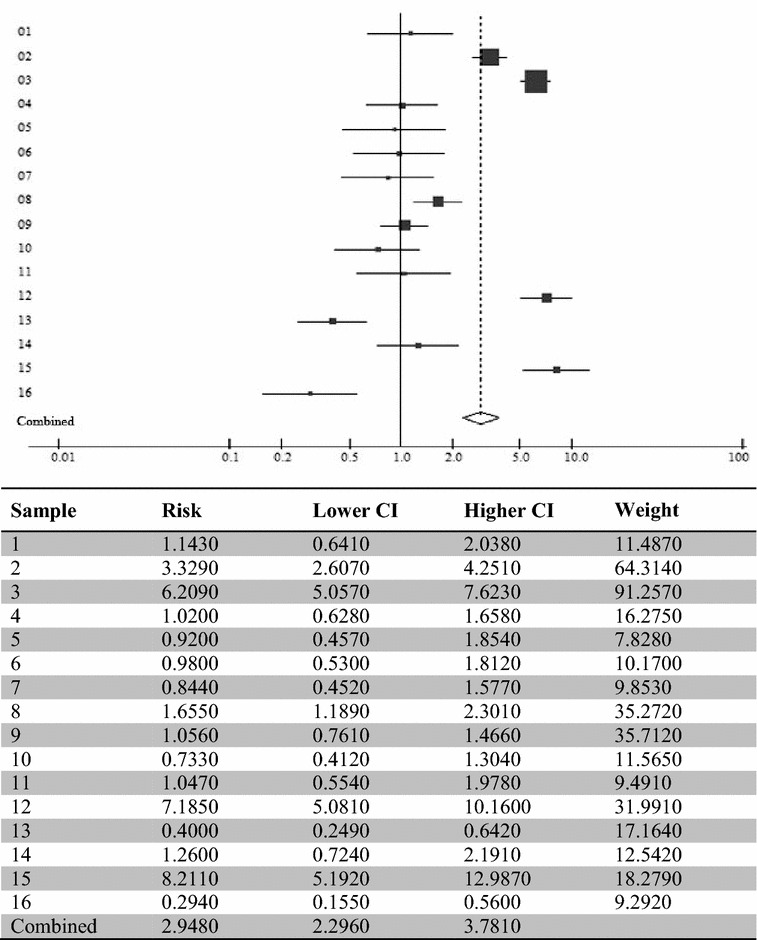
Meta-analysis of the suicidal ideation risk in children and adolescents with bipolar disorder. *CI* confidence interval

Based on the studies mentioned above, an adjusted calculation was performed of the joint risk of all studies to statistically evaluate the risk of a child or adolescent committing suicidal ideation when such factors are present. Figure 2 shows the result according to the analysis and in the order of studies from Table 2.

After analyzing 16 studies, a joint risk of 2.94 CI [2.29–3.78] was obtained. This means an almost three times higher risk of children and adolescents with BD developing suicidal ideation. A *p* value of 0.001 was obtained for the same analysis, which statistically shows the risk of a child or adolescent presenting the reported factors of developing suicidal ideation.

**Fig. 3:**
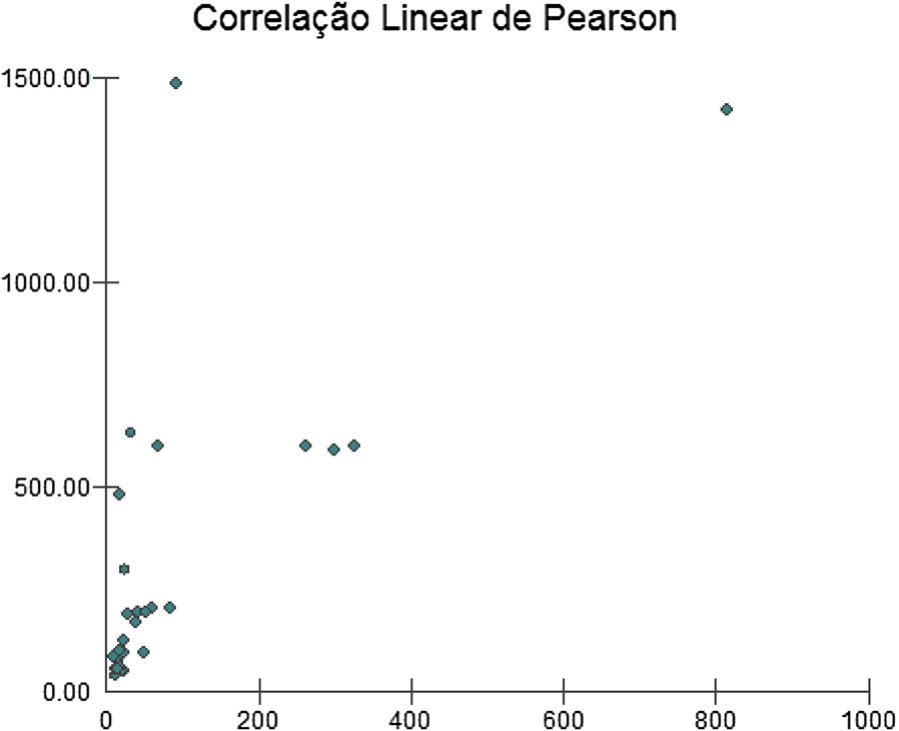
Pearson’s correlation

Figure 3 shows a statistical correlation calculation (Pearson) that was performed to evaluate the degree of statistical relation between the risk factors and the suicidal ideation phenomenon. The result can vary from −1 to +1, in which −1 is a perfect negative correlation, zero means no correlation, and +1 is a perfect positive correlation.

A significant correlation between the risk factors and the suicidal ideation was found as a result: *r* (Pearson) = 0.7103 and *p* < 0.001. Therefore, this fact confirms the idea that children and adolescents with BD are more vulnerable to suicidal ideation.

## Discussion

Many factors are present in children and adolescents with higher chances of developing suicidal ideation. Of the main ones, the presence of depressive symptoms more intensely predisposes the patient to developing the phenomenon [20]. These symptoms show relation with suicidal ideation (*p* < 0.05) when associated with characteristics like disincentive and pessimism and in the presence of BD, which is statistically significant [21].

When BD is associated with deficit of attention and hyperactivity, subjects present 2.38 more chances of developing suicidal ideation compared to those without the symptoms [22]. Some characteristics are statistically significant for the development of suicidal ideation, such as history of self-harmful behavior (OR = 2.45); psychiatric hospitalizations (OR = 2.48); mixed episodes (OR = 2.08); and psychosis (OR = 1.75) [23]. In general, the early beginning of BD in children and adolescents is associated with the increase of suicidal ideation risk if compared to the development in the adult phase [24].

When comorbidities are present with the BD in children and adolescents, the risk of suicidal ideation development increases [25, 26]. Psychiatric comorbidities are part of the main risk factors for the development of such phenomenon and therefore should be considered in the subject’s health evaluation [24, 26].

Family history of BD is an important risk factor for suicidal ideation, with a rate higher than 57% [18]. This family history relation is the basis of the theory of association with the presence of genes, since subjects with family history and genetic characteristics like the hypothalamic–pituitary–adrenal gene have 0.47 probability of developing suicidal ideation [27].

Other important relations are associations between suicidal ideation in BD and presence of fragile family relationships [28]. Higher rates of suicidal ideation are found in adolescents who live in an unfavorable family environment. Therefore, the focus of treatment is no longer directed only to the patient, but also to the family integrally [23].

If we analyze on the gender standpoint, females present a higher predisposal, especially if associated with depression symptoms, with a significance value of *p* < 0.005 [22]. Women have chances higher than 1.03 of developing suicidal ideation compared to men. More frequently when they face mixed episodes of the disease [29].

By making a joint analysis of the characteristics found in the studies, a risk of 2.94 with CI [2.29–3.78] and *p* value of 0.001 was found for the correlation between the analyzed characteristics and suicidal ideation. Thus, the association factors between BD and suicidal ideation in children and adolescents are statistically significant.

## Conclusion

Children and adolescents with bipolar disorder are statistically more vulnerable to the development of suicidal ideation. Many factors can influence the phenomenon like genetic factor, use of substances, comorbidity, age, female sex, emotional and physical negligence, sexual, emotional and physical abuse, and family factors.

Children that show factors associated with bipolar disorder have almost three times higher chances of developing suicidal ideation with a relation level above 70%, which is statistically significant. Continuous effort is still necessary to understand the factors of the association between BD and suicidal ideation in children and adolescents properly.

## Abbreviations

BAD: bipolar affective disorder
BD: bipolar disorder
ECA/USA: National Epidemiological Catchment Area Study
LILACS: Literatura Latino-Americana e do Caribe em Ciências da Saúde
OR: odds ratio
SciELO: Scientific Library Online
WHO: World Health Organization
